# Medical Society of the County of Erie

**Published:** 1911-03

**Authors:** John H. Pryor

**Affiliations:** Chairman


### SOCIETY PROCEEDINGS

### Medical Society of the County of Erie

### Report of the Committee on the "Division of Fees; and its Causes and Remedies."

JOHN H. PRYOR, M.D., Chairman.

AT a meeting of the Medical Society of the County of Erie, held February 21, 1910, Dr. M. D. Mann presented a paper entitled "Division of Professional Fees." The address was fully discussed and a resolution was unanimously adopted directing the president to appoint a committee to investigate the entire subject, including the causes and possible remedies. In obedience to such instructions a committee was appointed and respectfully reports as follows:

Frequent meetings have been held and the matter assigned us has received careful investigation and consideration. With the object of securing coöperation and information from the profession, a circular letter containing twelve questions was sent to each member of the Society and a reply requested. The response revealed disappointing apathy and lack of interest in problems which vitally affect the welfare, standing and ideals of the medical profession. About 540 circulars were sent and 31 replies received. Attention is simply directed to this demonstration of indifference and no comment ventured. The result of the inquiry revealed a practically unanimous agreement that the chief causes of commercialism and its attendant abuses were, overcrowding of the profession and too low a standard of education. Your committee has sought information wherever it could be obtained and has tried to arrive at definite conclusions after mature deliberation.

#### DR. MANN'S CHARGES SUSTAINED.

It has been found that Dr. Mann's statements and charges were true—that the practice of dividing fees or giving of commissions by some surgeons to physicians has existed in this city for several years, and that the exposure and criticism of the abuse was justified. We thoroughly approve acquainting the profession with the facts concerning this vicious and dangerous innovation, and favor warning the public of the unhappy results which will follow its continuance or increased prevalence.

#### SECRET METHODS EMPLOYED.

The division of fees has been accomplished by numerous methods. All of them are more or less adroit, deceitful and dishonest. The principal effort has been directed to provide secrecy. In the course of time some operators have become bolder than others and have gradually converted the practice of surgery into a traffic of operating on commission. No one publicly justifies the commercial bargain. If defended privately the excuse or argument is cynical, shifty, selfish or sophistical. After examination from every side there is no honest course except emphatic and unequivocal condemnation of this rather new species of hidden graft. No matter how cleverly the division of a fee is accomplished, it is done almost invariably without the knowledge of the patient. The person who pays for an operation does not known that part of the amount which has varied from 25 to 50% occasionally goes to the physician who recommended the operator. The physician and the surgeon are supposed to render their individual bills, and the afflicted person is entirely ignorant of the "gentlemen's agreement" or "community of interest" which has been introduced from the realms of high finance and legal honesty. The real purpose of the deal is to encourage the physician to send his patients where he can obtain a share of the money paid for relief or attempted relief. The surgeon may be highly competent or he may not and the physician may be influenced by financial encouragement. Anyway, the performance must pay because it has flourished and been profitable at times when other methods would probably have failed. At times, the demands of the physicians have been quite high and some of the prosperous merchants in surgery may begin to wonder if they have not created a Frankenstein.

PERNICIOUS RESULTS OF PRACTICE.

The practice may lead to unnecessary operating and junk surgery through increasing zeal to be busy and establish a false measure of success by the amount of income derived from business instinct and sagacity. The untrained and inexperienced cutter who has learned that there is money in operating, which is lost by the physician, is encouraged to obtain work which should go to the experienced, skillful surgeon who clings to the traditions and ideals of the profession; and will not cringe, stoop, or barter to obtain his earned and rightful privilege of employment. The fee may be increased or stretched by agreement to provide for distribution of the spoils, and altogether there is something about the whole wretched proceeding which smells of the rebater, the promoter, and the greed for disguised plunder.

MATTER SHOULD BE AIRED.

Some members of the profession keenly regret and reprobate any public exposure and discussion of the subject and fear that the public will become suspicious of all the members of the profession. The answer is simple: it is time the people knew of the practice and be given an opportunity to penetrate some of the mysteries surrounding the sick bed. In time the intelligently suspicious may distinguish between types of doctors and exhibit a tendency to investigate conditions quite puzzling today.

There is at least one profession that should be clean and have the confidence and trust of the public. Whatever may be its shortcomings in ability to help or save, the effort and purpose must be free from the taint of sordid commercial deals dependent upon human suffering and woe. There is very often no more complete picture of helplessness than the sick yearning for relief and not knowing where to seek needed succor. If abuses exist the profession must decide whether it will abolish them or allow them to prevail until the public is compelled to undertake the task. Your committee believes that the medical profession should perform the disagreeable work, and that an element is not afraid to expose or denounce iniquities which tend to degrade those who decently follow a noble calling.

### LOCAL SITUATION.

Honesty and a sense of duty compel us to call attention to the local aspects of this question and confess that an evil has existed. Surgeons can apply a prompt remedy if they will, by simply stopping the practice. The committee has learned with pleasure and gratification that practically all of the operators in this county have signed an agreement that they will not indulge in any division of fees, and that any violator of the agreement will submit to a penalty which may be fixed by the Erie County Medical Society. It remains for the Society to determine the value of this agreement and what steps shall be taken to ensure enforcement. The appointment of a committee to act as a court of honor, and consider complaints, examine evidence, and devise methods of punishment—when acts in violation of certain standards of professional dignity are perpetrated—is certainly worthy of careful consideration. We are sadly deficient in safeguards relating to professional conduct unless flagrant crime supplies a chance for decisive and wholesome action. The legal profession has more efficient control and well-defined, direct methods of procedure when they are employed. At present the purpose is to be suggestive in the hope of arriving at some consideration of this theme by the Society or its proper committee. If no other penalty can be found at this time, publicity in some form deserves attention as a possible corrective instrument. Something should be done to discriminate between the man whose influence is thoroughly damaging to the profession, and the one who helps to make it reputable and worthy of the highest admiration and confidence.

OVERCROWDED PROFESSION PRINCIPAL CAUSE.

Any study of the causes and possible remedies of forms of commercialism, and especially of the division of fees, must be considered in a broad way. The evils are not local but general, widespread and probably national in scope. Other cities and localities throughout the country report that the same conditions are prevalent, and have developed in recent years. It would be misleading and unjust to search for causes or seek for explanations in this region when we fully realise that we are dealing with an epidemic and not an endemic variety of infection. In classifying causes the chief factor seems to be the unfortunate and unnecessary overcrowding of the medical profession. Those who have studied this phase of the problem arrive at the conclusion that about one-third or at most, one-half of the present number of physicians and surgeons in the populous districts of this country, could fulfill all legitimate demands for human relief and secure a competent living. The average income is far below the amount required to permit of a mode of life consistent with the modern practice of medicine. A large proportion of the profession cannot obtain sufficient practice, experience or skill to become proficient. If the number of doctors in this country were diminished by two-thirds, or at least one-half, that proportion to the population would ensure adequate work and emolument and correspond more closely to conditions in other nations.

The lamentable overcrowding has a most deleterious effect upon the profession and the type of men who join its ranks. But the most baneful results will certainly be more keenly felt and appreciated by the public as they are discovered and better understood. At present this nation is in a semi-barbarous state as far as provisions for control of national health is concerned. The full meaning of the conservation of natural resources has not yet been recognised as including human beings. In considering overcrowding no attempt has been made to include the army of new pathies, faddists and variegated assortment of healers, pseudo-scientists, or the old contingent of perennial quacks and nostrum venders. Perhaps in the advance of preventative medicine and medical education we are wasting too much sympathy upon the class "who never considered it necessary to add the incident of learning to the accident of brains." It has been claimed that overcrowding has long existed, and that evidence of many tricks to secure advancement are comparatively recent. There is no time for judicious discussion of the question whether there has been a decline in the moral standards of the profession, and how much any change is due to imitation and the influence of business crookedness and predatory customs which abound in a favorable environment. The important thing is to call attention to the fact that conditions have changed which make the effects of overcrowding more acute, and the scramble for employment and a living more intense, and the temptation to resort to shrewd tactics more common and glaring.

### PROGRESS OF PREVENTIVE MEDICINE.

During the last twenty years, preventative medicine has made gigantic strides. The incidence of illness, particularly of childhood, has undergone a vast diminution, and the general death rate has practically been cut in half. The marvelous advance in surgery has removed a large group of patients from the field of medicine, and new discoveries have shortened the period of illness or changed its course. The tardy awakening to the importance of public health will add more and more force to the crusade against disease.

### CONTRACT PRACTICE AND OTHER EVILS.

Vicious, dangerous and cheap modes of practice have developed to a surprising extent in latter days. Medical and surgical relief under contract, and stultifying agreements with lodges, societies, benefit associations, etc., and underpaid services to life insurance companies, have demoralised practice among young men and robbed others of just remuneration. These abuses are largely indefensible, delusive to the patient and public as the results are mostly ineffective, the service superficial and careless, and often of no genuine value. It is only just to the young physician and the public that this increasing abuse should be investigated and fully considered at a future time. There is much harm and humbug in the practice, and the physician should no longer be a tool for crude, cheap work. Positions held by medical men almost invariably yield totally inadequate compensation. and any protest is unavailing because the supply is apparently unlimited. The young practitioner seeks opportunity for experience, and a chance to escape idleness while waiting for employment in a profession where there is little or no room. There are two classes —one seeking the sick to make a living, and another expecting a reference of a patient or a consultation.

Practice legitimately belonging to competent physicians, has been given over to faddists with a squint or kink, largely through the fault of narrow dogmatic members of the medical profession who could not or would not realise that the mentality of a patient required thought and attention, and that exercise of the body or its components was a physiological aid or necessity in treatment.

Hospitals and other institutions have been monopolised or exploited by a few, and some of the hospitals supported by philanthropy are simply hot-beds for fee splitting and commission jobbery. The industry is tolerated and winked at because the new method fills the private rooms. How much revenue can you supply, has occasionally become more essential than, how much ability and character can you offer, when appointments are considered by trustees, or dictated by the staff.

### GENERAL PRACTITIONER UNDERPAID.

The division of a fee is only one abuse. There are many others harder to perceive and reach, and some of them have given an impetus to this method of trading and may perpetuate it. Undoubtedly the inducement of a commission has been extended in a pernicious effort to compete and grasp a share of operative work. Again it has been used as a furtive evidence of sympathy toward the lesser paid physician. This leads to a consideration of one of the principal contributing factors related to superabundance of doctors and their fees. The cost of living has decidedly increased and the mode of life has undergone a transformation too little appreciated. The fee and the income have not changed in proportion, if at all. Extravagance is the fashion and the necessities of a progressive physician accumulates each year. He belongs to a class which is struggling along, surrounded by combinations and the waves of prosperity lose their force before reaching him.

The surgeon and the specialist have educated the public to place a higher value upon their services, and there is force in the contention as a rule. Special skill, experience, long training, responsibility and technic are required, and the qualified surgeon is rarely overpaid. Whether the surgeon who gives away half his fee regards himself as overpaid, is another question. The increase of operators and the lure of the knife because it loosens the purse strings, will soon equalise and distribute opportunity, and lower the rate of compensation. The surgeon has enjoyed halcyon days and deserved many of them, but he will have to guard and discipline the recruits to his guild, or the public will revolt.

### PHYSICIANS MUST DEMAND PROPER REMUNERATION.

The physician is actually and proportionately underpaid, and it is almost entirely his own fault. If overcrowding prevents the demand for proper remuneration because others will act for less, let him place the responsibility for the overproduction of doctors where it belongs and register his protest not alone for selfish reasons but vastly more, for the benefit of the whole profession and the community. The competition that affects livelihood is keenest and most demoralising among the mass and not among the few. There is no way by which the public can distinguish between the physician who has spent time and labor to become proficient and one who has not. We have but one degree and it may mean much or little. Nor is there any good reason why the fee of the physician should be rigidly fixed with no reference to the value of service. He should charge for the thoroughness, efficiency, skill, and time employed in his study of an individual case. Many times his diagnosis, advice and treatment are more valuable than surgical interference. The proper examination of a patient has become a problem involving time, wide knowledge, and chemical and microscopic analysis and search, requiring more and more special training and skill. The time has come for the physician to assert his position and claim what he deserves. He should receive his reward openly and not secretly, and resent undervaluation to the patient and not to the surgeon. Let him stand on his own feet and not beg or barter with the surgeon for a hidden share which he hasn't the courage to ask for. Let him seek assistance when he needs it as if he were the patient, and receive it with a clean hand from a clean hand and preserve a decent opinion of himself and his possibly more-fortunate confrere.

SUPERABUNDANCE OF MEDICAL COLLEGES.

The explanation of the overcrowding in the medical profession will be found in riveting attention upon the character of medical edѝcation in this country; and recently an opportunity has been afforded to reflect upon this interesting subject by the publication of the Carnegie Foundation Bulletin No. 4. It should be read by every member of this Society. This report may be considered intolerant and radical, but a great service has been performed; and the collection of facts based upon investigation will have a tremendous educational value and influence. Already its effect has become manifest, and the supplemental report will probably furnish a guide to action by comparison with European standards. It seems to be true that there are as many medical colleges in this country and Canada as in all the rest of the world. Canada still protects its population from the flood of graduates poured into this country. It also seems to be true that about one-third of the medical colleges in this country could be lifted to a higher modern standard and supply all the doctors needed for an indefinite time and growth of population. The facility and ease with which medical colleges could be established in this country has long been a disgrace. There probably was a time when the proprietary medical school with all its schemes for profits among a few, was tolerable or even seemed to be necessary. That time has passed and the school of the proprietary type and its self-created professors, will be eliminated. The medical school will ultimately be obliged to cease from depending upon students' fees for support, and liberal funds will be required to furnish the tuition which should be supplied today. The connection with a university will be close, true, and actual—not spurious or non-existant, and trustees and councils will probably cease to be rubberstamps or respectable, irresponsible nonentities, well described by Dickens.

### NEW STANDARD OF MEDICAL EDUCATION IMPERATIVE.

Many so-called medical colleges are still proprietary in spite of evasions and strenuous efforts to escape from that category. There are direct and indirect profits and scrutiny will go deeper and deeper to discover the true purpose and objects of these prolific institutions of learning. Is it not very strange that we have such a prodigal supply of medical colleges and that over-production of the graduates continues unabated, while the professors must know that the oversupply is unnecessary and can't be assimilated? Is it not perfectly plain that doctors are responsible for the superabundance of doctors, and the attendant evils which result from the wholly unnecessary excess? The most wholesome, natural remedy would follow an awakening of the whole profession to their interest and duty in dealing with a national disgrace. The united profession should decide upon the necessity for medical colleges, what status they should have and maintain, what shall constitute a medical education, and what shall be required of a licentiate to practise upon humanity. There should be control of the appointments of professors, and their duties and obligations to those really concerned should be defined. These matters should not be left to a few self-perpetuating and self-constituted faculties who have enjoyed too much power, unrestraint, and domination. National and state supervision and radical reorganisation of medical teaching and requirements, is imperative and inevitable.

There is no reason at present why the standard of qualificaton cannot be raised more rapidly, and the comparison with other countries made less apparent or glaring. The flood of graduates can be checked with safety. It is sophistical to claim that a better class will gradually replace those who are striving today. That is the old cry heard with every advance. The real demand is for increased intelligent and stringent legislation and some guarantee that a state license assures true proficiency.

### PRESENT STATE EXAMINATION NOT PROPER TEST.

A beginning has been made in this state by extending the power of the State Regents, and providing for a State Board of Medical Examiners. Objections and obstacles had to be overcome before this step was taken, and some good has been accomplished; but an examination for fitness to practise medicine and surgery by present methods must be obviously incomplete and unsatisfactory to anyone who has given the matter any careful thought. The Regents appoint the examiners. If any plan for selection because of special training or attainments, is employed, some of the results are truly surprising. A recent excellent appointment deserves warm commendation. The schools, dogmas, creeds, and sects are represented, and possibly the recommendatoin of candidates may emanate from medical societies during political sessions. The Board of Examiners is presumed to safeguard the public and act as a clearing house and check upon the medical colleges. When the graduate satisfies the examiners and the law, he is licensed to practise on anybody, anyway he chooses. The candidate is subjected to a written examination and is supposed by many to answer correctly 75 per cent. of the questions. As a matter of fact he is given fifteen questions, allowed to select ten, and must gain a marking of seventy-five upon them. Consequently he is really obliged to answer correctly 50 per cent. in accordance with the opinion of the examiners. The questions and answers are published frequently and it seems as though the students who make a collection of them and cram, or are quizzed assiduously, might find it advantageous. No time need be consumed in explaining to an intelligent physician how-crude, farcical and unreliable such a test for admission into one of the highest professions, must be of necessity. High percentages obtained in this way are cited with pride by the medical colleges as proof of superior teaching and preparation. The professors and the examiners are both anxious to advance the standards, but new obstacles and conservative policies seem to block the way.

NEW STANDARDS MUST REPLACE OLD.

Your committee has learned with gratitude that the time is now near when the candidates will be compelled to reach a percentage of seventy-five on answers to the full fifteen questions. Thus progress is gradually assured. The time seems to be ripe to insist that the state examinations should in reality prove a candidate's fitness to practise medicine and surgery by demonstration of his knowledge at the bed side and in the laboratory as well as by written evidence. Before entering such an examination, the applicant should be required to show that he has had actual experience and training in branches of medicine and surgery in a general hospital. It seems eminently fair to request that the licentiate to practise upon humanity in this state, should be as well qualified as the Government carefully provides for the sailor and soldier. If such competence could be required, there are a great many problems which would be effectually settled. Of course, the machinery would have to be changed. More money and a reorganisation would become necessary, but the real purpose in creating a State Board of Examiners would be achieved, and their function as sentinels fulfilled. The requirements in preliminary education should certainly be increased in this state.

Legislation should be secured if necessary to assist progressive action on the part of the Regents, and transform the personel, organisation and duties of the Board of Examiners in accordance with present conditions and the need of remedial measures. This society may as well lead in this direction and make its influence felt in the State Medical Society. It will take time and wisdom to sift the facts and arrive at safe conclusions. There are many interests involved, and while radical action is needed, it should be sane and practical. If there is need of reformation and a sincere desire for improvement, relief seems to lie along that path. If the manifold taints of commercialism are to be discouraged and decreased, and the tone of the profession is to be raised, paramount causes must be attacked. There may be full indulgence in garrulity and strong disapproval of wrongs expressed, but there are only two methods of gaining a greater height. The one who ought to climb must be helped or lift himself. It is about time to drive home the truth that "A little integrity is better than any career."

PUBLICITY RECOMMENDED.

Your committee unanimously recommends that this report be published in medical journals and copies be given to the daily press to be fully presented to the public if possible. Publicity is the safest, sanest course to follow. The confidence of the people must be maintained without equivocation or deception. If the revelation or confession hurts, let the blame rest where it belongs. The mass of the profession is sound, clean and unafraid to condemn ignoble motives and improper conduct which has stealthily stolen into its ranks. Regret is blended with the hope that frank disapproval will make any other action unnecessary.

FUNDAMENTAL CAUSES EXPLAINED.

The causes and effects seem easily discernible if they are fearlessly examined and the microscope is occasionally employed instead of a telescope. The historic course of events follow a natural sequence and it is not surprising that an avenging Nemesis is crying for retribution. When they are recited in proper order we can perceive a vast new nation with a rapidly increasing scattered population and no provision for the inception or control of medical education as it existed in older civilised countries. Medical colleges were of necessity created by small practically self-appointed groups with little or no restraint. Later they were too often established and employed for personal aggrandisement, questionable advantage, and direct and indirect profits. The false system inculcating special privileges, spread without proper jurisdiction or supervision. In the course of time the outcome was a multitude of proprietary medical schools and gradually a vast superabundance of diploma factories and an oversupply of the product. Substantial benevolent returns from the alumni in proportion to the annual crop, were most enticing. Then came control and domination of institutions and increase of lieutenants. Nothing paid so well as professorships and titles when used to promote the reference of cases and consultations. Frequently the aim in supply was quantity, not quality. Ultimately, lamentable overcrowding and a struggle for existence became only too evident. Ambitious doctors endeavored to advance and seize a share of reputation and compensation. Competition was difficult and became keener and fiercer. Many means were suggested to check the flood of graduates and a state examination was introduced. The hoped-for benefit has not been obtained. The number of unnecessary colleges and professors, the yearly flux of deluded graduates, the overcrowding and the baneful competition, continued to exist. Then came the increased cost of living and the added expense of modern practice with no increase and often a decrease in income. This condition is tremendously influenced by industrial and financial revolutions accompanied by a toleration for moral obliquity and censurable methods in business enterprise. At last a vicious, misdirected mode of competition is found which proves financially successful. It is devoted largely to a pitiful scramble for the dollar and is still limited to a small part of the profession. One mode of gaining ascendancy and its tribute, was followed by another much less tolerable and more reprehensible from a moral standpoint. It is one symptom of reckless revolt, and an adaption of the policy "After us the deluge." The great mass of the profession has a right to protest and complain of the character and amount of competition to which they have long submitted, but the fee splitter and the schemer by any method only add another more dangerous incubus. It is the outcropping of the worst polluted with a desire to substitute one abuse for another of which many have become thoroughly tired and exasperated.

The blame for the degradation and turmoil in the profession should be traced to its true origin and the labor of reformation belongs largely to those who are responsible for the conduct and out-put of institutions ostensibly intended for ethical and medical education and the laxity of government control which is the core of the whole problem. Let there be no privilege not beneficial to the whole profession and a fair field on level ground. The cleansing process should extend beyond any one evil now exposed to the light. Its associates and their ancestors need ventilation and disinfection also.

REMEDIES SUGGESTED.

As the work of the committee progressed, many cynical remarks have been heard to the effect that any exposure and consequent action will prove ephemeral and futile. It is claimed that this society has no legal power to check or abolish such an evil as has been described. But there are methods which can be used in a drastic manner, if necessary. We ask that this report shall not fall cold and be deposited quietly in the archives of this society. What the Erie County Medical Society begins should be thoroughly finished. A form of bribery must be starved by ostracism and denunciation, or strangled by some form of punishment. Let the matter be kept before the society at future meetings. Investigation should continue. Activity and determined persistence will encourage other societies to follow, and the benefit will be widespread. Now that the facts are known and the dangers appreciated, reform or supine toleration are the only courses left open.

RECOMMENDATIONS OF COMMITTEE.

To ensure and possibly guide effective remedial efforts, the following recommendations are submitted and approval requested:

1. Publication of this report in the medical journals and the daily press.

2. Reference of that portion relating to complaint, investigation and devising some form of punishment, to the committee on "Professional Relations," and thus provide for continued watchfulness and further consideration.

3. It is recommended that the secretary transmit a communication to the State Board of Regents, urging the necessity of a higher preliminary educational requirement, and definite changes in the method and scope of the examination for a license to practise in this state; and that this matter be referred to a proper standing or special committee to arouse interest, stimulate inquiry and promote necessary progressive action leading to higher medical education.

4. It is also recommended that a special committee be appointed to report at an early date upon the extent, character, effects of professional services under contract or by agreement with companies, corporations, fraternal societies and life insurance companies; this report to include if possible, practical remedies which may be applied to diminish this form of employment or place it upon a different basis.

Your committee was assigned a difficult, unpleasant and undesired task, and has discharged a duty with honest intent, free from any malice, prejudice or unkind feeling.

Respectfully submitted,

JOHN H. PRYOR, M.D. (Chairman),
M. D. MANN, M. D.,
BERNARD BARTOW, M. D.,
F. PARK LEWIS, M. D.,
WILLIAM GAERTNER, M. D.,
IRVING P. LYON, M. D.,
E. A. BOWERMAN, M. D.,
T. J. WALSH, M. D.,
GROVER WENDE, M. D. Ex-officio.

